# Deletion of endogenous Tau proteins is not detrimental in *Drosophila*

**DOI:** 10.1038/srep23102

**Published:** 2016-03-15

**Authors:** Sylvie Burnouf, Sebastian Grönke, Hrvoje Augustin, Jacqueline Dols, Marianna Karina Gorsky, Jennifer Werner, Fiona Kerr, Nazif Alic, Pedro Martinez, Linda Partridge

**Affiliations:** 1 Max Planck Institute for Biology of Ageing, Joseph-Stelzmann-Strasse 9b, 50931 Cologne, Germany; 2 CECAD Cologne Excellence Cluster on Cellular Stress Responses in Aging Associated Diseases, 50931 Cologne, Germany; 3 Institute of Healthy Ageing, Department of Genetics, Evolution and Environment, University College London, Darwin Building, Gower Street, London, WC1E 6BT, UK

## Abstract

Human Tau (hTau) is a highly soluble and natively unfolded protein that binds to microtubules within neurons. Its dysfunction and aggregation into insoluble paired helical filaments is involved in the pathogenesis of Alzheimer’s disease (AD), constituting, together with accumulated β-amyloid (Aβ) peptides, a hallmark of the disease. Deciphering both the loss-of-function and toxic gain-of-function of hTau proteins is crucial to further understand the mechanisms leading to neurodegeneration in AD. As the fruit fly *Drosophila melanogaster* expresses Tau proteins (dTau) that are homologous to hTau, we aimed to better comprehend dTau functions by generating a specific *tau* knock-out (KO) fly line using homologous recombination. We observed that the specific removal of endogenous dTau proteins did not lead to overt, macroscopic phenotypes in flies. Indeed, survival, climbing ability and neuronal function were unchanged in *tau* KO flies. In addition, we did not find any overt positive or negative effect of dTau removal on human Aβ-induced toxicity. Altogether, our results indicate that the absence of dTau proteins has no major functional impact on flies, and suggests that our *tau* KO strain is a relevant model to further investigate the role of dTau proteins *in vivo*, thereby giving additional insights into hTau functions.

Dysfunction of the neuronal microtubule-associated Tau protein is observed in several age-related neurodegenerative disorders including Alzheimer’s disease (AD), where the accumulation of highly post-translationally modified Tau species leads to the formation of intracellular neurofibrillary tangles and subsequent neuronal death. To date, no treatment for AD has been developed and approved that could efficiently halt the neurodegenerative process together with preventing, delaying or curing cognitive decline. Hence, many efforts are needed to further understand the biology and physiological functions of Tau on one hand, and the mechanisms leading to Tau dysfunction and toxicity on the other.

The fruit fly *Drosophila melanogaster* has been widely used as a powerful model organism to investigate *in vivo* the toxicity of proteins linked to neurodegenerative diseases, including AD (for reviews see^1^,^2^). Noteworthy, most of these models rely on ectopic expression of human transgenes. While these systems are suitable to investigate toxic gain-of-function of proteins, they present limitations regarding the study of physiological functions or the analysis of loss-of-function effects. Therefore, to gain some insights into the biology of human proteins, one approach is to discover the function of their homologue proteins in other species. Interestingly, the human Tau protein has a fly homologue called *Drosophila* Tau (dTau)^3^, which also displays microtubule-binding properties^3^,^4^. Some studies have focused on dTau functions using either *Drosophila* RNAi lines^5^,^6^,^7^ or *tau* hypomorphic/deficiency lines^4^,^5^,^8^,^9^, leading to contradictory results regarding the potential detrimental or beneficial effects of *Drosophila tau* removal. While these fly lines constitute a first interesting approach to evaluate dTau functions, one drawback is that they can lead to off-target effects or disrupt the expression of other genes than *tau*.

To circumvent these problems with specificity of lowered *tau* expression and to further dissect the functions of the endogenous dTau protein, we have generated by homologous recombination a *tau* knock-out (KO) fly line that presents a specific deletion of the genetic region spanning from exon 2 (E2) to exon 6 (E6), which codes for the microtubule-binding region of dTau. Using this line, we observed that the constitutive deletion of endogenous dTau proteins did not trigger major effects in terms of fly survival and fly climbing ability. In addition, we could not detect any detrimental effect of *Drosophila tau* removal on the transmission of nerve impulse within the *Drosophila* giant fiber system. Finally, our results show that toxic effects on fly survival related to the expression of human Aβ in the adult *Drosophila* nervous system are not mediated by dTau. Altogether, our study suggests that the specific deletion of *Drosophila* Tau proteins is neither detrimental nor beneficial for flies at the macroscopic level.

## Results

**Figure 1:**
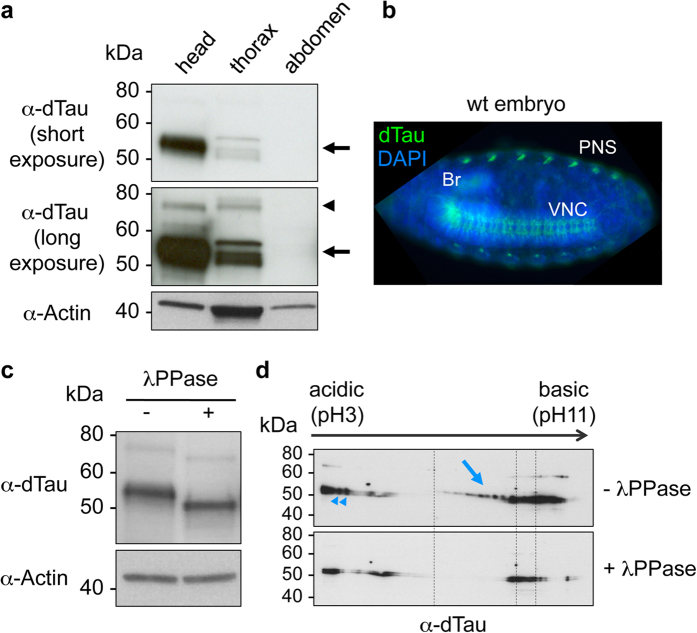
Characterisation of the *Drosophila* Tau protein. (**a**) Western blot analysis showed an enrichment of dTau proteins in heads and thoraces of wild-type flies, with several isoforms being detected. The major dTau isoforms migrated at an apparent MW of ±55 kDa (arrow) while a less abundant isoform, mainly detected following a long-time exposure, was detected at ±75 kDa (arrowhead). No dTau signal could be detected in extracts from fly abdomens. The Actin control confirmed the presence of proteins in all investigated samples including fly abdomens. (**b**) Immunofluorescence on wt *Drosophila* embryos showed that dTau (green) was localised in the fly nervous system (Br: brain, VNC: ventral nerve chord, PNS: peripheral nervous system). DAPI (blue) was used to stain cell nuclei. (**c**,**d**) Phosphatase treatment on fly head extracts led to a shift of dTau apparent MW for both the 55 and 75 kDa bands (**c**) and to the loss of several dTau-positive spots (arrow and arrowheads) as observed by 2D gel electrophoresis (**d**) suggesting that they corresponded to phosphorylated dTau species. Actin is shown as a loading control (**c**).

In order to characterise fly Tau proteins, we first performed Western blot analysis on wild-type (wt) *Drosophila* heads, thoraces and abdomens (Fig. 1a). dTau proteins were particularly abundant in fly heads, with a major dTau isoform running at the apparent molecular weight (MW) of ±55 kDa (arrow, Fig. 1a). Longer exposure time allowed the detection of a less-abundant dTau isoform displaying a higher apparent MW (±75 kDa, arrowhead, lower panel of Fig. 1a). Fly thoraces also contained dTau proteins, although in lower amounts than fly heads. Interestingly, we also observed several dTau isoforms in extracts from fly thoraces, presenting a slightly different profile from head dTau isoforms. No dTau signal could be detected from fly abdomens (Fig. 1a). In addition, staining of dTau (green, Fig. 1b) in wt *Drosophila* embryos showed an enrichment of dTau proteins in the fly nervous system including the brain (Br), the ventral nerve chord (VNC) and the peripheral nervous system (PNS).

Interestingly, we observed that dTau proteins were highly phosphorylated. Indeed, following incubation of wt fly head extracts with a lambda phosphatase (λPPase), we observed a shift of the apparent MW of dTau proteins (Fig. 1c) using an anti-total-dTau antibody. Further, we performed a two-dimensional (2D) gel electrophoresis analysis to evaluate the isoelectric profile of dTau proteins and better delineate the extent of dTau phosphorylation^10^. Interestingly, many dTau isovariants were observed upon staining with an anti-dTau antibody, suggesting that dTau was highly post-translationally modified (Fig. 1d). While most of dTau isovariants were found in a basic pH range, a few dTau spots were also detected in a very acidic pH range. To determine whether some of these isovariants corresponded to phosphorylated dTau species, we performed a λPPase treatment on head protein extracts prior to 2D gel electrophoresis. This treatment triggered the partial loss of dTau immunoreactivity, both in the basic (Fig. 1d, arrow) and the acidic (Fig. 1d, arrowheads) pH range, indicating that these spots consisted of phosphorylated dTau species. Altogether, these experiments highlight that dTau proteins are phosphorylated and enriched in the fly nervous system, similarly to human Tau proteins.

**Figure 2:**
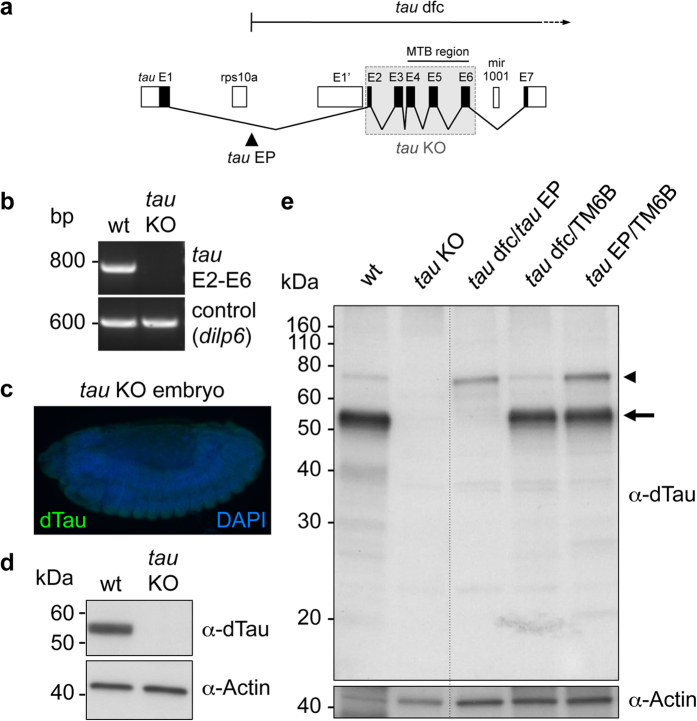
Generation of a *Drosophila tau* KO line. (**a**) Simplified representation of the *Drosophila tau* gene based on Flybase data for *tau*-RA/RB transcripts, and the surrounding annotated genes. E1’ is indicated as an alternative exon that is part of the *tau*-RG transcript. Exons 2 to 6 of the *tau* gene, including the microtubule-binding (MTB) region, were removed by homologous recombination. The insertion locus of *tau*^*EP3203*^ (*tau* EP) and the deleted region observed in the *tau* dfc (Df(3R)MR22) line are shown. (**b**) RT-PCR for both the *tau* gene (E2-E6) and a control gene (*dilp6*) were performed in wt and *tau* KO fly samples. No transcript could be observed for *tau* at the expected size in the *tau* KO line. (**c**) Immunofluorescence on *tau* KO *Drosophila* embryos showed no detectable signal for dTau (green). DAPI (blue) was used to stain cell nuclei. (**d**) Western blot analysis from heads of wt and *tau* KO flies showed no signal for the 55 kDa dTau band in the *tau* KO line. Actin is shown as a control for comparable protein levels. (**e**) Long-time exposure for dTau staining showed no detectable bands in the *tau* KO line. The *tau* dfc/*tau* EP line did not show any staining for the 55 kDa dTau band (arrow) but the higher 75 kDa isoform (arrowhead) was detected in this line. The relative amounts of dTau-75 kDa were increased compared to the dTau-55 kDa in the heterozygous *tau* EP/TM6B line but not in the heterozygous *tau* dfc/TM6B line. Actin is shown as a loading control. A dashed line delineates cut parts from the same western blot membrane and film.

To further decipher the functions of endogenous dTau proteins in *Drosophila*, we performed homologous recombination to generate a *tau* knock-out (KO) line presenting a selective deletion of the E2 to E6 region of the *tau* gene (exon numbering based on FlyBase data for *tau*-RA transcript), hence avoiding mutation of surrounding genes such as *rps10a* and *mir-1001* (Fig. 2a). Specific deletion of *Drosophila tau* was verified by RT-PCR (Fig. 2b), using primer pairs targeting E2 and E6 of *tau*. At the protein level, we observed no detectable signal for dTau in *tau* KO *Drosophila* embryos using immunofluorescence (Fig. 2c). Similarly, no signal could be detected by western blot for dTau in the *tau* KO line, despite comparable protein levels as shown using an anti-Actin antibody (Fig. 2d). Interestingly, while both the 55 kDa and 75 kDa dTau isoforms were absent from the *tau* KO line (Fig. 2e), we observed that flies carrying a combination of *tau* P-element insertion (*tau*^*EP3203*^, referred to as “*tau* EP”) and *tau* deficiency (Df(3R)MR22, referred to as “*tau* dfc”) alleles, still expressed the 75 kDa dTau isoform (“*tau* dfc/*tau* EP”, Fig. 2e, arrowhead; see Fig. 2a for insertion/deletion sites). It appeared that this was the consequence of the P-element insertion in the first *tau* intron, as we observed an up-regulation of the 75 kDa dTau as compared to the 55 kDa dTau isoform in extracts from heterozygous *tau*^*EP3203*^ flies (“*tau* EP/TM6B”, Fig. 2e). Conversely, the 75 kDa-dTau protein was expressed at normal levels in heterozygous *tau* dfc flies (“*tau* dfc/TM6B”, Fig. 2e). Importantly, we observed that the pattern of dTau expression in a *Drosophila* mutant specifically lacking both *tau* E1 and the associated promoter region was similar to that of the *tau* dfc/*tau* EP line (“*tau* ∆E1”, Supplementary Fig. 2a,b). Indeed, while we detected no signal for dTau corresponding to the 55 kDa isoform in the *tau* ∆E1 fly line, the 75 kDa dTau was still expressed (Supplementary Fig. 2b).

**Figure 3:**
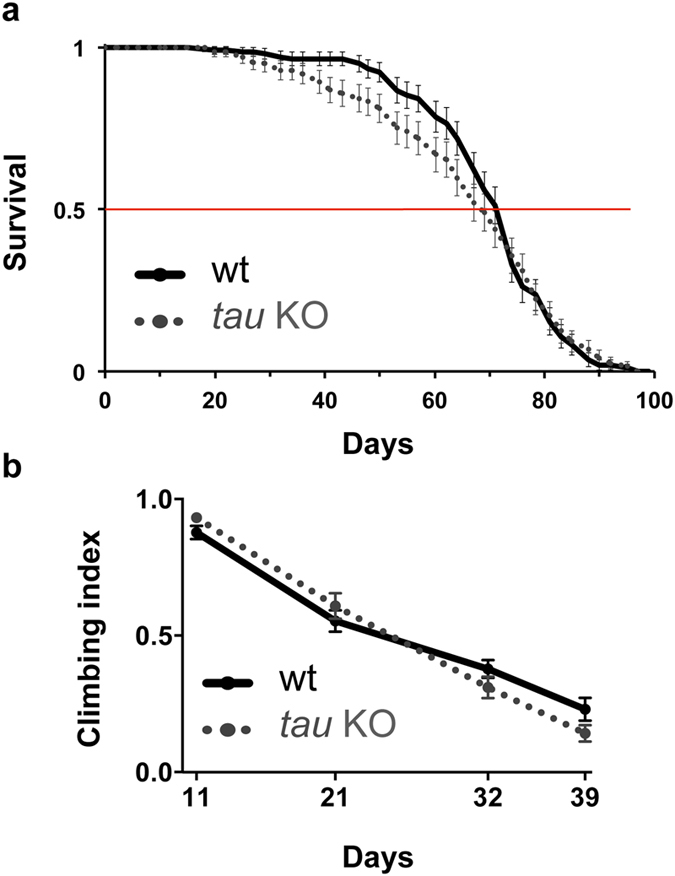
Knocking out *Drosophila tau* did not affect fly lifespan or locomotion. (**a**) Survival curves of wild-type (plain line) and *tau* KO (dotted line) flies were comparable (p > 0.05, log-rank test). (**b**) Climbing ability was evaluated in flies aged for 11 to 39 days. No climbing defect could be observed in *tau* KO flies as compared to wt flies (p > 0.05, two-way ANOVA).

Noteworthy, no signal for dTau was detected in *tau* KO flies (Fig. 2e). We therefore used this line to investigate the effect of knocking out *tau* on fly survival and fly climbing ability (Fig. 3). Interestingly, we did not observe any major effect of dTau removal, either on fly lifespan (Fig. 3a, p > 0.05, log-rank test) or on climbing behaviour (Fig. 3b, p > 0.05, two-way ANOVA).

**Figure 4:**
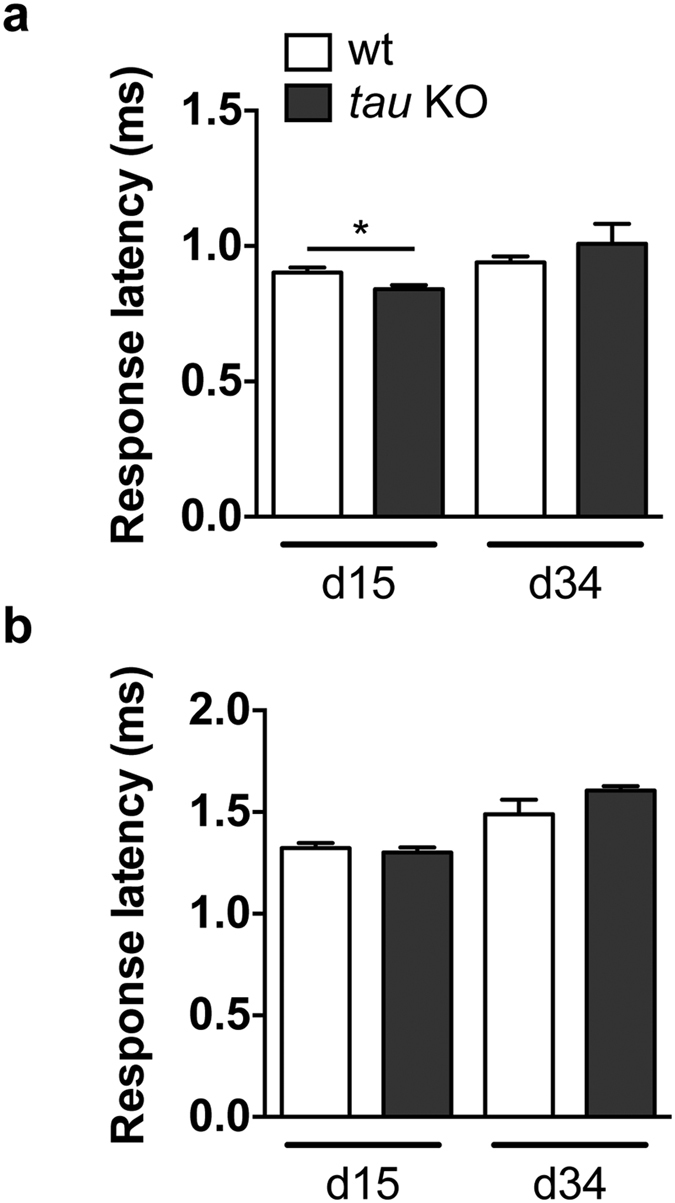
Knocking out *Drosophila tau* did not impair neurotransmission in the giant fiber neuronal system. Electrophysiological recordings were performed in 15- and 34-day-old wt and *tau* KO flies. Following stimulation of the giant fiber, response latency, the time for the electric signal to reach the TTM (**a**) and the DLM (**b**) was measured (*p < 0.05, Student’s t-test, n = 4–6/genotype).

Given that dTau is a neuronal protein, we hypothesized that its removal could impair neuronal fitness and function. We therefore performed electrophysiology experiments on adult *Drosophila* to quantitatively assess the functional status of the giant fiber pathway^11^ in *tau* KO compared to wt flies. To do so, following electric stimulation of the giant fiber in the fly brain, we measured the time (response latency) for the electric signal to either reach the tergotrochanteral muscle (TTM, Fig. 4a) or the dorsal longitudinal muscle (DLM, Fig. 4b). Surprisingly, we did not detect any detrimental effect of dTau removal on response latencies in either branch of the circuit (Fig. 4). dTau removal even led to an apparent beneficial effect in 15-day-old *tau* KO flies, with a shorter response latency in the TTM, though this effect was not observed in older ages (*p < 0.05, Student’s t-test, Fig. 4a).

**Figure 5:**
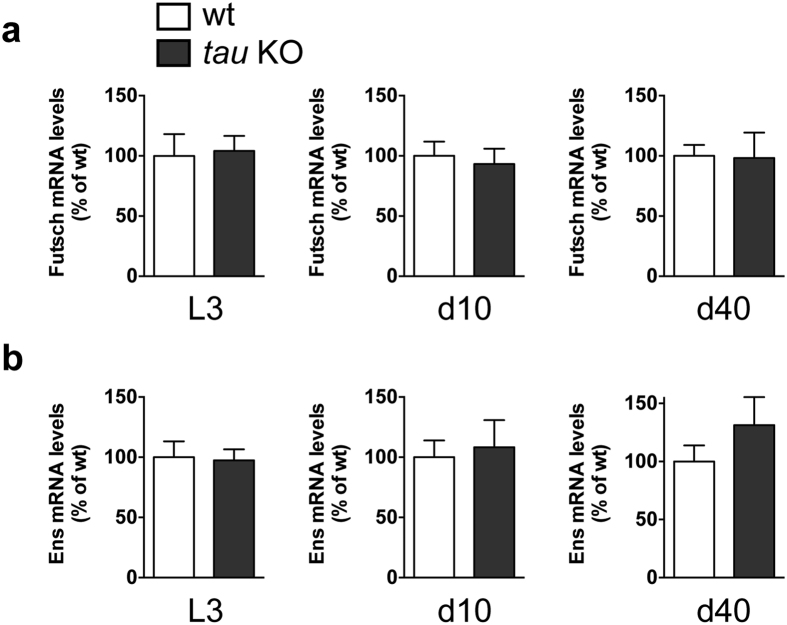
Knocking out *Drosophila tau* did not trigger compensatory effects in other MAPs transcript levels. qPCR evaluation of *futsch* (**a**) and *ensconsin* (**b**) mRNA levels was performed in third instar larvae and in 10- and 40-day-old wt and *tau* KO flies. *Actin5c* was used for normalisation. No difference in expression could be detected among the lines (p > 0.05, Student’s t-test, n = 4–6 replicates/genotype).

Given the lack of detrimental effects of dTau removal observed on fly survival, locomotion and neuronal function, we evaluated whether the *tau* KO line had developed functional compensation through up-regulated expression of other fly microtubule-associated proteins, i.e. *futsch*, which is the only known MAP1 homologue in flies, and *ensconsin,* a MAP7 homologue that is abundant in the fly nervous system. We measured mRNA levels of *futsch* (Fig. 5a) and *ensconsin* (Fig. 5b) during fly development (third instar larval (L3) stage) and in head extracts from young and old (10 and 40 days old, respectively) wt and *tau* KO flies, but we could not detect any significant up-regulation of their levels in the absence of dTau (p > 0.05, Student’s t-test, Fig. 5).

**Figure 6:**
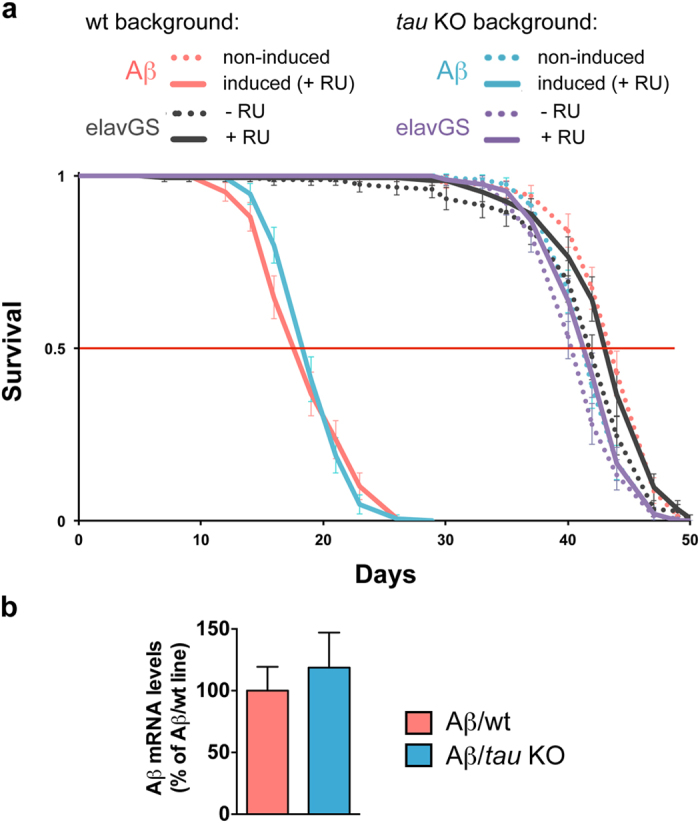
Knocking out *Drosophila tau* did not affect toxicity of human Aβ for fly lifespan. (**a**) Adult-onset expression of human Aβ in fly neurons (elavGS driver) led to a drastic reduction of fly survival in both wt (Aβ/wt, plain red line) and *tau* KO (Aβ/*tau* KO, plain blue line) backgrounds (p > 0.05, RU-induced Aβ/wt vs. RU-induced Aβ/*tau* KO, log-rank test). Non-induced controls are shown as colour-matched dotted lines. RU486 feeding did not impair survival of the elavGS driver control lines (black and purple lines). (**b**) qPCR analysis of Aβ mRNA levels retrieved from head extracts of RU-induced Aβ/wt and Aβ/*tau* KO flies showed no difference in expression (p > 0.05, Student’s t-test, n = 4–5/genotype).

Finally, in the context of the amyloid cascade hypothesis, we investigated whether dTau removal influenced the toxicity induced by human Aβ in the fly. While the over-expression of human Aβ in the adult fly nervous system, using the inducible neuronal elavGS-Gal4 driver, was highly detrimental for fly survival (“Aβ/wt” plain red curve, Fig. 6a), we did not observe any significant modulation of Aβ-induced toxicity in the *tau* KO genetic background (“Aβ/*tau* KO”, plain blue curve, p > 0.05 vs. induced-Aβ/wt, log-rank test, Fig. 6a). Importantly, we controlled for Aβ transcript levels by qRT-PCR in both induced-Aβ/wt and induced-Aβ/*tau* KO lines and did not observe any difference (p > 0.05, Student’s t-test, Fig. 6b). These results therefore indicate that the removal of dTau did not affect Aβ-induced toxicity, suggesting that dTau proteins do not mediate Aβ-induced toxicity for fly survival.

Altogether, our results suggest that the complete lack of endogenous dTau proteins is not detrimental for *Drosophila* in terms of survival, locomotion or neuronal function and does not modulate the toxicity of human Aβ for fly lifespan.

## Discussion

Deciphering the physiological functions of the Tau protein is essential to further understand the pathophysiological mechanisms leading to its dysfunction and aggregation in neurodegenerative disorders such as Alzheimer’s disease. Using a new *Drosophila* line specifically lacking endogenous dTau proteins, we demonstrated in the present study that dTau removal was not detrimental for fly survival, climbing ability or neuronal function. In addition, our results indicated that the lack of endogenous dTau proteins did not modulate the toxic effects induced by Aβ expression in the adult fly nervous system.

*Drosophila* Tau and human Tau are homologous proteins that present amino acid sequence similarities and share functional properties, notably in terms of microtubule binding^3^,^4^. Besides this, one of hTau’s major features is phosphorylation. Indeed, the longest hTau isoform of the central nervous system, which consists of 441 amino acids, holds 85 putative phospho-sites (for review see^12^). Similarly, the 361 amino acid dTau protein (corresponding to the most abundant Tau-PA/Tau-PB protein isoforms on FlyBase) contains 52 putative phospho-sites (32 serines, 17 threonines and 3 tyrosines), suggesting that dTau proteins might also be modified and regulated by phosphorylation events. In support of this hypothesis, it was previously shown that dTau proteins could be detected by a cross-reacting antibody raised against phosphorylated hTau at both Ser262 and Ser356 (12E8 antibody^13^), indicating that dTau proteins were phosphorylated at this epitope. In line with these observations, we observed, using a combination of lambda phosphatase treatment, 1D- and 2D-gel electrophoresis, that dTau proteins were highly phosphorylated in fly heads under physiological conditions. Understanding the specific effects of phosphorylation events for dTau functions, e.g. binding to microtubules, subcellular localisation or protein-protein interactions, will require further experiments.

While originally described as relatively simple^3^, the complexity of the *Drosophila tau* gene locus was revealed by more recent observations (Flybase data, summed up in Supplementary Fig. 1). Indeed, dTau has different promoters and is subjected to alternative splicing, leading to the production of several *tau* transcripts and dTau protein isoforms. Thirteen *tau* transcripts have been annotated so far, 12 of which hold the microtubule-binding region (MTBR) that is encoded by exons 4, 5 and 6. Importantly, one of the latter, *tau*-RA, encoding the 361 amino acid Tau-PA isoform, is expressed at high levels and represents approximately 80% of all *tau* transcripts^14^. Western blot analysis of the recombinant Tau-PA protein revealed an apparent MW of 50–55 kDa (data not shown), suggesting that the major 55 kDa-dTau band that we observed in fly heads is constituted, at least in part, of the Tau-PA isoform. In addition, using the *tau* ∆E1 *Drosophila* line specifically lacking *tau* exon 1 and the associated promoter region, we did not detect the 55 kDa-dTau isoform, further supporting that this dTau isoform held *tau* exon 1. Interestingly, we observed the residual expression of a higher dTau isoform with the apparent MW of 75 kDa in the *tau* ∆E1 fly line. Similarly, expression of this dTau isoform was observed in both *tau* dfc/*tau* EP and *tau* EP/TM6B fly lines, suggesting that this was a feature of the P-element insertion in the first *tau* intron. Noteworthy, an alternative *tau* promoter driving the expression of the *tau*-RG transcript was recently annotated (Flybase data, see Supplementary Fig. 1). This promoter is located downstream of both the *tau* sequence that is excised in the *tau* ∆E1 mutant and of the P-element insertion in the *tau* EP line, suggesting that its expression is not blunt in those lines. Altogether, this suggests that the alternative downstream *tau* promoter is driving the expression of the bigger 75 kDa-dTau isoform, which likely corresponds to the Tau-RG protein.

To generate the *tau* KO *Drosophila* line, we specifically excised exons 2 to 6 of *Drosophila tau*, thereby ensuring that expression of the 12 known MTBR-holding transcripts would be abolished. A small *tau*-RE transcript may still be expressed in the *tau* KO line, potentially leading to the production of a 10 kDa Tau-PE polypeptide (Supplementary Fig. 1). Though deciphering whether this isoform is normally expressed in fly neurons will require further experiments, the fact that this polypeptide does not contain any MTBR however suggests that it does not bind to microtubules.

Further, using homologous recombination to generate the *tau* KO line, we preserved embedded genes such as *rps10a* and *mir-1001* by restricting the genetic deletion to *tau* sequence. Therefore, this strategy ensured the unbiased analysis of dTau functions, preventing, on one hand, residual expression of some microtubule-bound dTau isoforms and, on the other, preserving the presence of surrounding genes, as their alteration might confound the interpretation of results regarding dTau functions.

The genetic fly tools so far available to investigate dTau loss-of-function are based on either P-elements insertions or excisions to disrupt *tau* expression, or on expression of RNAi constructs to decrease *tau* levels. These models, though giving some hints with regards to dTau functions, should be considered carefully. Indeed, insertion of P-elements such as *tau*^*EP3203*^ in the first intron of *tau*^9^ did not prevent the expression of some dTau isoforms (Fig. 2e) and has also been reported to alter *rps10a* expression (Flybase data). As for deficiency lines, their generation relies on the imprecise excision of P-elements. In the case of the Df(3R)MR22 (*tau* dfc) line, the recombination of *tau*^*EP3203* ^9 produced a deletion of 62 kb within the fly genome, which was homozygous lethal and affected genomic regions beyond the *tau* gene, spanning approximately 16 kb^3^. Finally, *tau* RNAi lines only partially reduce *tau* levels^7^ and might also induce off-target effects.

Therefore, we aimed to investigate the consequences of the specific removal of *Drosophila* Tau proteins using our *tau* KO fly line. Interestingly, we did not observe any major effect of dTau removal on fly survival, climbing ability or neuronal fitness. These results are in line with previous studies investigating the consequences of knocking-out mouse Tau (mTau) proteins, where homozygous *tau* KO mice were viable and presented no overt phenotype^15^,^16^,^17^,^18^. It was however observed that older *tau* KO mice developed motor deficits including reduced performance on the Rotarod and altered locomotion in the open field test^19^,^20^. In contrast, *tau* KO flies displayed no overt locomotion defects throughout life as observed using a counter-current climbing system. Even though we cannot rule out that basal locomotion might be altered in *tau* KO flies, it appeared that dTau proteins did not regulate the locomotion of flies in response to a mild stress induced by a mechanical jolt.

Interestingly, the absence of major detrimental effects observed following *tau* removal occurred without any change in the expression of *futsch* and *ensconsin*. These two microtubule-associated proteins are enriched in the fly nervous system^21^,^22^ and constitute the only known fly homologues of MAP1 and MAP7, respectively. In *tau* KO mice, even though MAP1A, MAP1B and MAP2 levels were not altered in adult brains, MAP1A expression was increased in newborn mice, presumably to compensate for the loss of mTau^15^,^16^,^17^. In contrast, we did not observe any difference in the transcript levels of the fly MAP1 homologue *futsch*, either at the larval stage or in adult flies, suggesting that the absence of overt phenotypes observed following dTau removal was independent from any compensatory feedback loop involving *futsch* in flies. However, Futsch loss-of-function was shown to alter dendritic and axonal growth^23^ during fly development, suggesting that Futsch is crucial for neuronal development. In addition, other reports indicate that neuronal over-expression of dTau in *futsch* mutants could partially rescue some of the detrimental phenotypes induced by Futsch loss-of-function^21^. Further experiments will be needed to determine whether dTau removal exacerbates the phenotypes observed in *futsch* loss-of-function fly mutants.

Finally, in the context of the amyloid cascade hypothesis, we investigated whether dTau proteins mediated the toxicity of human Aβ in *Drosophila*. Many studies have highlighted beneficial effects of absence of endogenous mTau proteins with regards to human Aβ toxicity^24^,^25^,^26^,^27^, indicating that mTau mediated Aβ toxic effects. However, mTau removal was also reported to exacerbate Aβ-induced cognitive alterations in older mice^28^, indicating that additional studies are needed to understand the complexity of the interaction between mTau proteins and human Aβ peptides. Importantly, we did not observe any modulation of Aβ-induced toxicity in *tau* KO flies, their survival being similar to that of flies expressing human Aβ in a wild-type genetic background. This result suggests that dTau proteins were not mediating human Aβ toxicity for fly lifespan. However, previous results have suggested that dTau loss-of-function (*tau*^*EP3203*^/Df(3R)MR22) could reduce toxicity induced by Aβ expression in adult fly neurons^8^. Though the implication of other genes than *tau* cannot be firmly excluded regarding this effect in the latter line, one could speculate that the 75 kDa-dTau isoform, the expression of which is up-regulated in the *tau* EP/*tau* dfc line, displays some particular function that would be beneficial in particular condition such as stress conditions triggered by human Aβ expression. This aspect will deserve further investigation.

In summary, we have generated a new *Drosophila tau* KO line specifically lacking endogenous dTau proteins and avoiding potential side effects that could result from the alteration of other genes. Removal of endogenous dTau proteins did not lead to overt detrimental or beneficial effects on survival, climbing ability or neuronal function in *Drosophila*. Further investigations can be achieved using this new fly line to understand the role of dTau proteins and thereby give some insights into human Tau functions.

## Material and Methods

### Generation of the *Drosophila tau* KO and *tau* ∆E1 lines by homologous recombination

We used ends-out homologous recombination^29^ to generate two different mutant *Drosophila tau* alleles named *tau* ∆E1 and *tau* KO. The sequences for *tau* promoter and exon 1, and *tau* exons 2 to 6, respectively, were replaced by a *white*^*hs*^ marker gene (Fig. 2a and Supplementary Fig. 2a). In order to clone the *tau* donor constructs for homologous recombination, approximately 4 kb of the 5′ and the 3′ flanking sequences were PCR-amplified using the BAC clone CH321-16D24 (BACPAC Resource Center, Oakland, California, USA) as template. For the *tau* ∆E1 donor construct, primers SOL192/SOL193 and SOL298/SOL299 were used to amplify the 5′ and 3′ arms, respectively. For the *tau* KO donor construct, primers SOL196/SOL197 and SOL198/SOL199 were used to amplify the 5′ and 3′ arms, respectively. Subsequently, PCR products were sub-cloned into the pGX attP vector^30^ using the indicated restriction sites and were full-length sequenced. The pGX attP vector was obtained from the *Drosophila* Genomics Resource Center (Bloomington, Indiana, USA). The *tau* donor constructs were transformed into the germ line of *Drosophila melanogaster* by P-element-mediated germ line transformation using the Best Gene *Drosophila* Embryo Injection Service (Chino Hills, California, USA). Crosses for ends-out homologous recombination were carried out according to the rapid targeting scheme^31^. Subsequently, the *white*^*hs*^ marker gene was genetically mapped and homologous recombination events were identified by PCR.

Primers used for the *tau* KO line:

SOL196: TAGCGGCCGCGACCAAGGGAAGCCAACCAAAAG (NotI)

SOL197: TAGGTACCTTAAATGGCTTCACTAATGGTCTTTGG (KpnI)

SOL198: TAGGCGCGCCATGTGTTCCCGAAGGTGTTTG (AscI)

SOL199: TACTCGAGCACGATTCCCTCCCGCAACA (XhoI)

Primers used for the *tau* ∆E1 line:

SOL192: TAGGTACCGCTAACTGCCCTTTTTGGACGCTT (KpnI)

SOL193: TAGCGGCCGCGCAGATTCGTGGCAACAA (NotI)

SOL298: TAACTAGTGCATACGCCGCGTGTGCTTTAG (SpeI)

SOL299: TAGGCGCGCCTGCAACAACCCAGCGAAGAGC (AscI)

### Fly stocks and maintenance

All fly stocks were kept at 25 °C on a 12:12 h light:dark cycle at constant humidity and fed with standard sugar/yeast/agar medium (15 g/L agar, 50 g/L sugar, 100 g/L yeast, 30 mL/L nipagin and 3 mL/L propionic acid).

Human Aβ_1−42arctic_ (E22G mutation) bearing a secretion signal from the *Drosophila* necrotic gene was synthesized by MWG Operon (Germany) using insect optimized codons and was cloned into the pUASTattB vector for insertion into the attP40 landing-site locus of the fly genome using a φC31-mediated integration. Flies expressing one copy of the Aβ_1−42arctic_ gene were used for experiments. The neuron-specific Elav-GeneSwitch-Gal4 inducible driver line (elavGS) was derived from the original elavGS 301.2 line^32^ and obtained as a generous gift from Dr. H. Tricoire (CNRS, France). Expression using the elavGS driver was achieved through addition of RU486 (Mifepristone) to fly food at a final concentration of 200 μM, while food from control flies was supplemented with the vehicle, ethanol. All lines were backcrossed into a white Dahomey (wDah) wild-type, outbred genetic background for at least ten generations prior to experiments. The newly-generated *tau* KO and *tau* ∆E1 lines were homozygous viable and were therefore kept as homozygous stocks. *tau* dfc (Df(3R)MR22; stock 9530) and *tau*^*EP3203*^ (stock 17098) lines were obtained from Bloomington *Drosophila* stock centre.

All experiments were carried out with female flies and at the temperature of 25 °C except electrophysiology recordings in Fig. 4 and lifespans in Fig. 6, which were performed at 29 °C.

### Lifespan analysis

For lifespan experiments, 150 to 200 once-mated, female flies per genotype were allocated to lifespan vials at a density of 10 flies per vial. The number of dead flies was recorded every 2 to 3 days while flies were transferred to fresh food. Lifespan results are expressed as the proportion of survivors ±95% confidence interval.

### Climbing assay

Climbing assays were performed blindly throughout the life of flies as described before^33^,^34^. At least 3 replicates of 20 females flies per genotype were used for the experiments.

### Generation of the anti-dTau antibody

The full-length (FL, 1.1 Kb) *tau* (CG31057) region was amplified from a pUAST-*dtau* vector (kind gift from Efthimios Skoulakis, Vari, Greece^35^) using the following primers: FL forward, 5′-CACCATGGCGGATGTCCTGGAG-3′; FL reverse, 5′-tgcatttcggcatTTAGCTTTGTTGA-3′ (lower case denotes region in pUAST vector). FL-*dtau* cDNA was then cloned into pENTR/D-TOPO entry and pDEST17 expression vectors, according to the manufacturers’ instructions (Invitrogen), and confirmed by sequencing. For dTau recombinant protein expression and subsequent antibody production, BL21-DE3 cells (Invitrogen) were transformed with mini-prepped (Qiagen) FL-*dtau* cDNA, and induced by incubation with 1 mM IPTG (Isopropyl β-D-1-thiogalactopyranoside). Recombinant proteins were purified, using a Nickel-NTA column, by Fast protein liquid chromatography (AktaPurifier, GE Healthcare) across a 25–400 mM imidazole gradient. dTau-containing fractions were TCA-precipitated and gel-purified by SDS-PAGE, after which bands were excised and sent for antibody production (Eurogentech). Rabbit SK6427 anti-dTau antibody was used for subsequent experiments.

### Immunofluorescence on *Drosophila* embryos

This procedure was based on Kaczynski and Gunawardena^36^ with some modifications. Briefly, following removal of the chorion and fixation in a 2% formaldehyde solution, embryos from wt and *tau* KO flies were incubated for one hour in blocking solution (5% BSA in PBT containing 0.2% of Triton X-100) at 4 °C and subsequently incubated overnight at 4 °C with the anti-dTau antibody (1/1000 in PBT with 5% BSA). Alexa Fluor 488 secondary antibody was used at a dilution of 1/200 in PBT with 5% BSA for two hours at 4 °C. Embryos were then mounted in Vectashield with DAPI mounting medium and visualised using a LEICA DMI4000B/DFC 340FX inverted microscope and a 10× objective.

### Electrophysiology measurements

Recordings from the Giant Fiber circuit were made essentially as described in Allen *et al.*^37^ and Augustin *et al.*^11^ and initially described by Tanouye and Wyman^38^. Briefly, flies were anaesthetized by cooling on ice and secured in wax, ventral side down, with the wings held outwards in the wax. A tungsten wire (ground electrode) was placed into the abdomen, and tungsten, stimulating electrodes were pushed through the eyes and into the brain to deliver a 40 V pulse for 0.03 ms using an npi electronic ISO-STIM 01D stimulator. Recordings were made from the tergotrochanteral muscle (TTM) and the contralateral dorsal longitudinal muscle (DLM) using glass microelectrodes (resistance: 40–60 MΩ). The electrodes were filled with 3 M KCl and placed into the muscles through the cuticle. Responses were amplified using Axoclamp 900A microelectrode amplifier (Molecular Devices, USA) and the data digitized using an analogue-digital Digidata 1440A digitizer (Molecular Devices, USA) and Axoscope 10.5 software (Axon Instruments, USA). For response latency recordings, at least 3–5 single stimuli were given with a 5 s rest period between each stimulus. Recordings were performed blindly from at least 4 flies per genotype.

### Sample preparation and Western Blotting

10 to 20 female heads, thoraces or abdomens were homogenized by sonication in 100 μL of ice-cold RIPA buffer supplemented with Complete mini without EDTA protease inhibitor (Roche). Protein concentration was measured using the BCA protein assay kit (Pierce) according to the manufacturer’s instructions. 10 μg of total proteins were supplemented with 2× LDS containing reducing agent (Invitrogen) and heated at 100 °C for 10 minutes prior to loading on 10% Bis-Tris Criterion gels (Biorad). Proteins were transferred to 0.45 μm nitrocellulose membranes (GE Healthcare) that were subsequently blocked in TNT buffer (Tris–HCl 15 mM pH 8, NaCl 140 mM, 0.05% Tween) with 5% non-fat dry milk for 1 h at room temperature and incubated overnight at 4 °C with antibodies recognizing either full-length dTau (1/10,000) or β-Actin (1/200,000, Abcam). HRP-conjugated anti-mouse or anti-rabbit antibodies (1/10,000, Invitrogen) were used for 1 h at room temperature and detection was performed using an ECL chemiluminescence kit (GE Healthcare) and Hyperfilms (GE Healthcare).

### Phosphatase treatment

Heads of wild-type female flies were homogenized by sonication in ice-cold RIPA (Pierce) buffer supplemented with Complete mini without EDTA protease inhibitor (Roche). Fifty micrograms of total proteins were treated with Lambda Phosphatase (New England Biolabs) for 3 hours at 37 °C. Twenty units of enzyme per μg of total proteins were used. Samples were stored at −80 °C until use for 1D and 2D gel electrophoresis.

### 2D gel electrophoresis

2D gel electrophoresis experiments were performed based on previous reports^39^ with some modifications. Fifty micrograms of proteins from fly heads were precipitated using the 2D clean-up kit (GE Healthcare). The resulting pellet was resuspended in 2D buffer (7 M urea, 2.5 M thiourea, 4% CHAPS) by sonication and supplemented with 0.5% IPG buffer 3–11 (GE Healthcare), 1% destreak (GE Healthcare) and traces of Bromophenol blue. Following overnight incubation with the protein samples in an IPG box (GE Healthcare), rehydrated Immobiline Drystrips (pH 3–11 NL, 11 cm) were placed into the Ettan IPGphor 3 Isoelectric Focusing Unit (GE Healthcare) and covered with mineral oil. The isoelectrofocalisation was performed following the manufacturer’s instructions, after which the strips were incubated in equilibration buffer (6 M urea, 30% glycerol, 2% SDS, 50 mM Tris-HCl pH 6.8, traces of bromophenol blue) supplemented first with DTT (10 mg/mL) for 15 min and with iodoacetamide (50 mg/mL) for another 15 min. Strips were then placed on top of 10% Bis-Tris IPG+1 Criterion gels (Biorad) for SDS-PAGE and proteins were subsequently transferred to 0.45 μm nitrocellulose membranes for antibody staining.

### RNA extraction and RT-PCR

Total RNA was extracted from heads of female flies (20–25 heads per replicate) using a Trizol-Chloroform-based procedure (Invitrogen). One microgram of RNA was then subjected to cDNA synthesis using the SuperScript Vilo Mastermix (Invitrogen). PCRs were performed on 4 μL of cDNA using the HotStar Taq Plus polymerase (Qiagen) and the following primers: ACAATGACAGCGGCGTGGATG (*tau* E2 forward), CTTCTCACCGCCACCGGGC (*tau* E6 reverse), GAAAAGGAAAGGAAGCGCCG (*dilp6* control primer forward) and GGGTGTGGCTGAGTGGTGG (*dilp6* control primer reverse).

### Quantitative real-time PCR

Following treatment with DNAse I (Ambion), 300 ng of RNA were subjected to cDNA synthesis using the SuperScript Vilo Mastermix (Invitrogen). Quantitative real-time PCR was performed using TaqMan primers (Applied Biosystems) in a 7900 HT real-time PCR system (Applied Biosystems). *Actin5c* was used as a normalization control and the relative expressions of *futsch*, *ensconsin*, and Aβ were determined by the ΔΔ*C*_T_ method. Four to six independent biological replicates per group were analysed.

### Statistical analysis

For lifespan experiments, statistical differences were assessed using the log-rank test. Other results are expressed as mean ± sem and differences between mean values were determined using either Student’s *t* test or two-way ANOVA, using Graphpad Prism software. *p* values < 0.05 were considered significant.

## Additional Information

**How to cite this article**: Burnouf, S. *et al.* Deletion of endogenous Tau proteins is not detrimental in *Drosophila. Sci. Rep.* **6**, 23102; doi: 10.1038/srep23102 (2016).

## Supplementary Material

Supplementary Information
