# Supplementary material for: Deletion of endogenous Tau proteins is not detrimental in Drosophila
Source: Sci Rep. 2016 Mar 15;6:23102. doi: 10.1038/srep23102 (PMC4792132; doi:10.1038/srep23102)

Deletion of endogenous Tau proteins is not detrimental in *Drosophila*

Sylvie Burnouf[1], Sebastian Grönke[1], Hrvoje Augustin[2], Jacqueline Dols[1], Marianna Karina Gorsky[1], Jennifer Werner[1], Fiona Kerr[2], Nazif Alic[2], Pedro Martinez[2] and Linda Partridge[1,2]*

[1]Max Planck Institute for Biology of Ageing, Joseph-Stelzmann-Strasse 9b, 50931 Cologne, Germany; CECAD Cologne Excellence Cluster on Cellular Stress Responses in Aging Associated Diseases, 50931 Cologne, Germany.
[2]Institute of Healthy Ageing, Department of Genetics, Evolution and Environment, University College London, Darwin Building, Gower Street, London, WC1E 6BT, UK.

*Correspondence should be addressed to L. P. (Linda.Partridge@age.mpg.de; Telephone: 0049(0)221-37970602; Fax: 0049(0)221-3797088602).

SUPPLEMENTARY FIGURE LEGENDS

Supplementary Figure 1. Description of the *Drosophila tau* gene, *tau* transcripts and dTau proteins (adapted from Flybase). This map depicts all the current known information about the fly *tau* gene, its annotated transcripts and polypeptides. Coding sequences are indicated in orange and non-coding sequences in grey. This information was extracted and adapted from Flybase (FB2016_01, released January 14, 2016).

Supplementary Figure 2. Analysis of a *tau* ΔE1 *Drosophila* mutant. a. Simplified representation of the *Drosophila tau* gene and the surrounding annotated genes. The *tau* ΔE1 fly line was generated by homologous recombination (indicated in blue) and bears a specific deletion of *tau* exon 1 and promoter region. Downstream regions of *tau*, including the *tau* E2 to E7 sequences and *tau* E1', which contains an alternative *tau* promoter region driving expression of the *tau*-RG transcript, were not affected in this mutant line. **b.** Western blot analysis from heads of *tau* ΔE1 flies showed a dTau signal for the 75kDa dTau isoform (arrowhead) but not for 55kDa isoform (arrow), similarly to what was observed for the *tau* dfc/*tau* EP line. Actin is shown as a loading control. A dashed line delineates cut parts from the same western blot membrane and film. Both dTau and Actin blots were expanded from Figure 2e.

Supplementary Figure 1. Description of the *Drosophila tau* gene, *tau* transcripts and dTau proteins (adapted from Flybase)

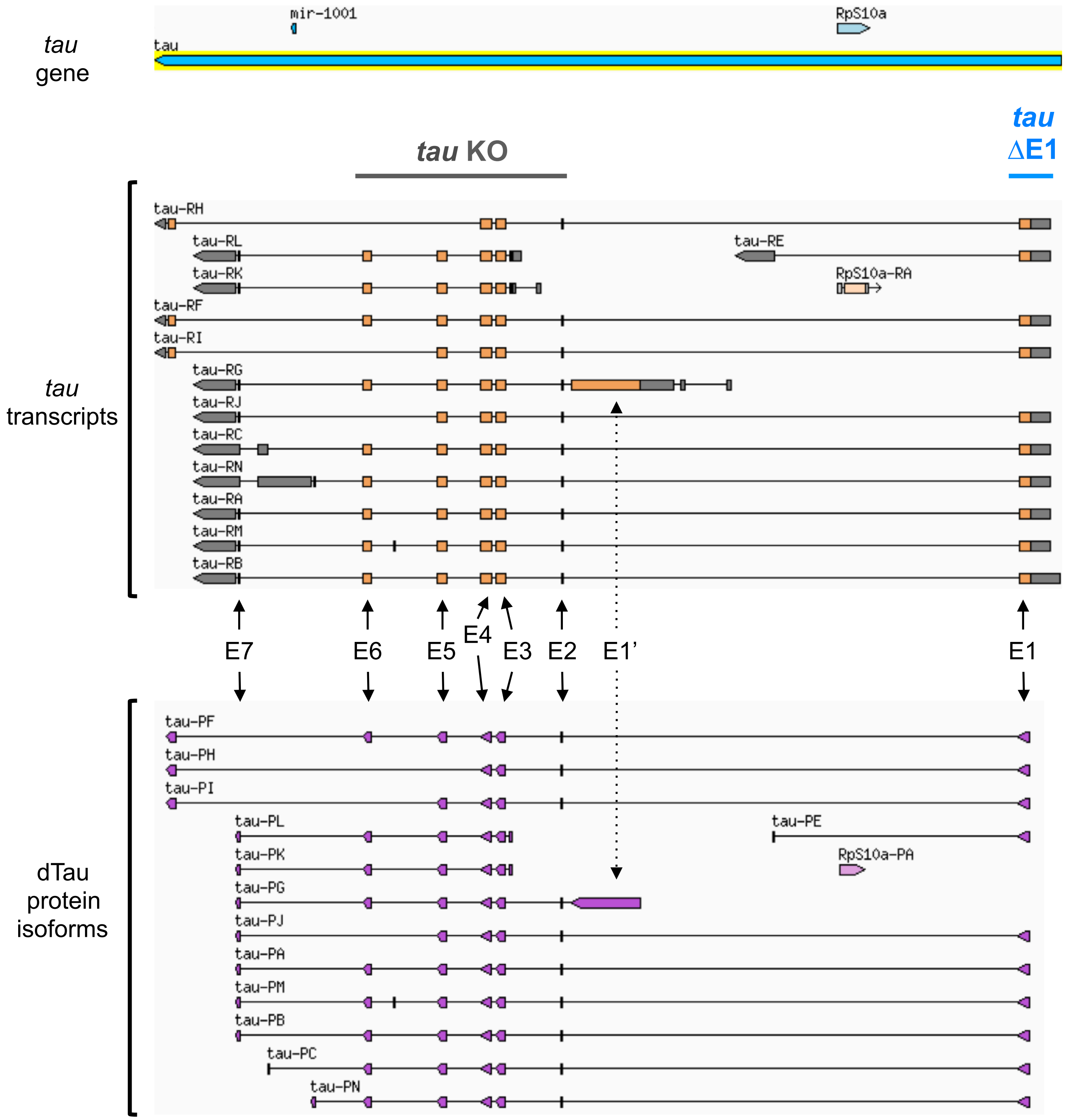

Supplementary Figure 2. Analysis of a *tau* ΔE1 *Drosophila* mutant

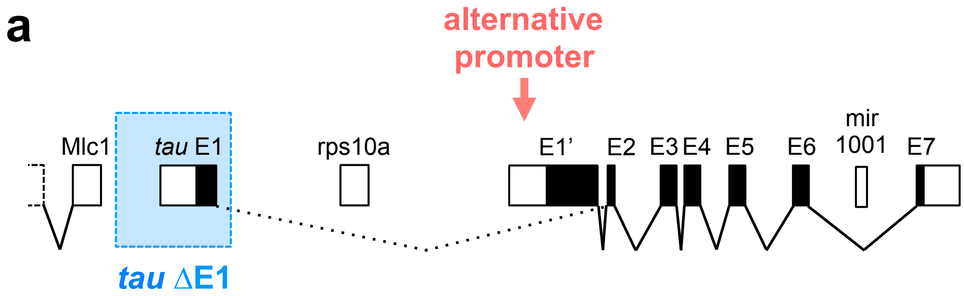

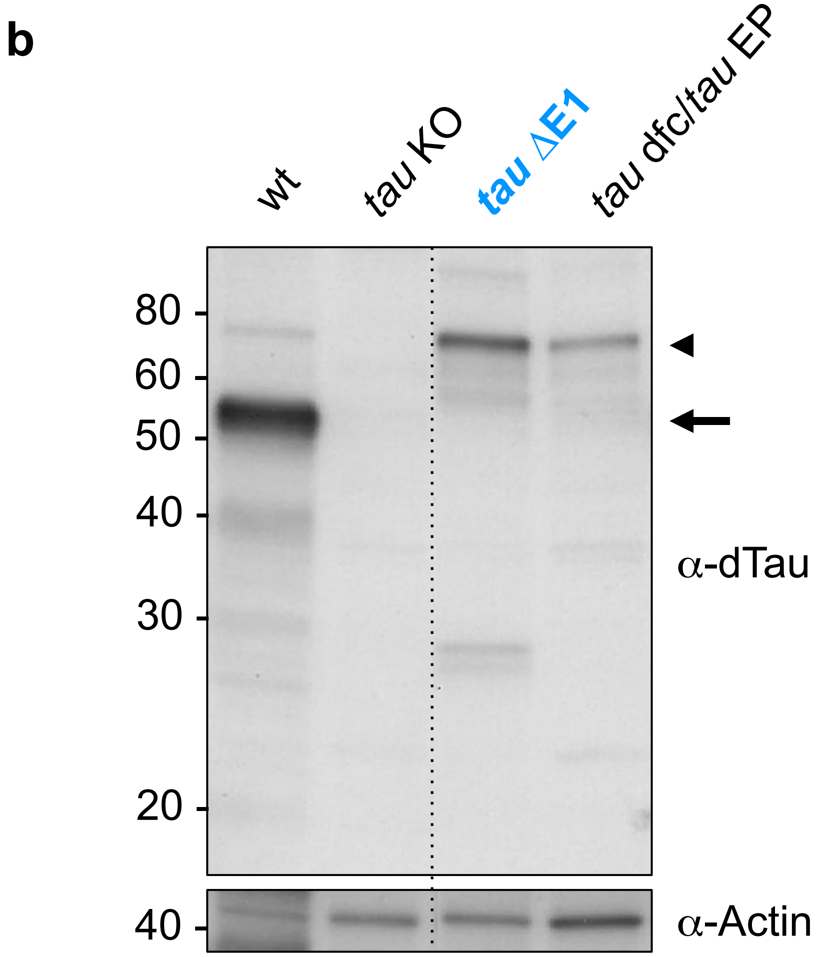

Supplement: Supplementary Information [file srep23102-s1.pdf]
